# GABALAGEN Facilitates Pentobarbital-Induced Sleep by Modulating the Serotonergic System in Rats

**DOI:** 10.3390/cimb46100663

**Published:** 2024-10-04

**Authors:** Minsook Ye, Kyoung-min Rheu, Bae-jin Lee, Insop Shim

**Affiliations:** 1 Department of Physiology, College of Medicine, Kyung Hee University, Seoul 02447, Republic of Korea; jh486ms22@naver.com; 2 Marine Bioprocess Co., Ltd., Busan 46048, Republic of Korea; kmin.rheu@gmail.com (K.-m.R.); hansola82@hanmail.net (B.-j.L.)

**Keywords:** GABALAGEN, electroencephalography (EEG), sleep, pentobarbital-induced sleep model, sedative-hypnotic activity, GABA

## Abstract

Gamma-aminobutyric acid (GABA) is one of the inhibitory neurotransmitters with beneficial effects including sedative properties. However, despite various clinical trials, scientific evidence regarding the impact on sleep of orally ingested GABA, whether natural or synthesized through biological pathways, is not clear. GABALAGEN (GBL) is the product of fermented collagen by *Lactobacillus brevis* BJ20 (*L. brevis* BJ20) and *Lactobacillus plantarum* BJ21 (*L. plantarum* BJ21), enriched with GABA and characterized by low molecular weight. The aim of this study was to investigate the effect of GBL on sleep improvement via a receptor binding assay in a pentobarbital-induced sleep-related rat model. We utilized a pentobarbital-induced sleep-related rat model to conduct this research. The present study investigated the sedative effects of GBL through electroencephalography (EEG) analysis in the pentobarbital-induced sleep animal model. Exploration of the neural basis of these positive effects involved evaluating orexin in the brain via immunohistochemical methods and 5-HT in the serum using an enzyme-linked immunosorbent assay (ELISA). Furthermore, we conducted a binding assay for 5-HT_2C_ receptors, as these are considered pivotal targets in the mechanism of action for sleep aids. Diazepam (DZP) was used as a positive control to compare the efficacy of GBL. Results: In the binding assay, GBL displayed binding affinity to the 5-HT_2C_ receptor (IC50 value, 5.911 µg/mL). Administration of a low dose of GBL (GBL_L; 100 mg/kg) increased non-rapid eye movement sleep time and decreased wake time based on EEG data in pentobarbital-induced rats. Administration of a high dose of GBL (GBL_H; 250 mg/kg) increased non-rapid eye movement sleep time. Additionally, GBL groups significantly increased concentration of the 5-HT level in the serum. GBL_H decreased orexin expression in the lateral hypothalamus. Conclusion: Overall, the sedative effect of GBL may be linked to the activation of serotonergic systems, as indicated by the heightened affinity of the 5-HT_2C_ receptor binding and elevated levels of 5-HT observed in the serum. This suggests that GBL holds promise as a novel compound for inducing sleep in natural products.

## 1. Introduction

Sleep is an essential aspect of human life. Worldwide, a significant number of people suffer from sleep disorders, with insomnia being the most commonly reported sleep disturbance. In Europe, up to 10% of the adult population experiences sleep disorders [1]. Since insomnia becomes more common as people age, this issue becomes particularly concerning in countries like China with growing older populations [2]. Insomnia is a sleep disorder characterized by difficulty falling asleep or staying asleep [3]. Sleep disturbances can lead to mental dysfunction, daytime sleepiness, and various health and socioeconomic challenges [4]. People who have been dealing with insomnia for extended periods may also face depression and a diminished quality of life [5]. The classes of drugs commonly used for the treatment of insomnia include γ-aminobutyric acid type A (GABAA)-benzodiazepine (BZD) receptor agonists, selective serotonin reuptake inhibitors, melatonin receptor agonists, antidepressants, and antihistamines [6]. However, insomnia is a common side effect of many medications [7]. Efforts to find an ideal sedative-hypnotic that provides better results without side effects are still ongoing. On the other hand, natural sleep aids and herbal remedies, which can improve sleep quality without side effects, are increasingly used as alternatives to prescription drugs for the treatment of insomnia [8].

The primary focus for the molecular target in sedative activity has centered on the regulation of the serotonergic (5-HT) system, which is implicated in diverse mental functions associated with relaxation, stress, and sleep regulation [9]. The 5-HT_2_ receptor (comprising subtypes 5-HT_2A_, 5-HT_2B_, and 5-HT_2C_), conventionally known to be coupled with G-proteins, has been identified as a key player in various central nervous system functions, including mood regulation, sleep modulation, and satiety [10]. Specifically, the 5-HT_2C_ receptor has been highlighted as a crucial therapeutic target in addressing insomnia [11].

GABA is an amino acid that occurs naturally in the human body and is considered to be the primary inhibitory neurotransmitter in the central nervous system of mammals [12]. GABA plays a crucial role in maintaining mental health [13]. It is involved in various physiological processes such as increasing brain protein synthesis, elevating growth hormone concentration, lowering high blood pressure, regulating diabetes, and facilitating diuresis. GABA has a calming effect and is potentially useful in treating insomnia and depression [14].

Previous studies have shown that people with acrophobia exposed to high altitudes experienced increased alpha-brain waves and reduced levels of immunoglobulin A [15,16] after consuming 100 mg of GABA, which contributed to relaxation and reduced anxiety [17]. Additionally, studies have demonstrated that taking 100 mg of GABA can significantly reduce sleep latency and non-rapid eye movement (NREM) sleep latency, making it easier for patients to fall asleep quickly [18]. Given its bioactive nature and physiological functions, there is a growing interest in the production and comprehensive analysis of GABA. Biosynthetic strategies have predominantly centered on lactic acid bacteria (LAB), primarily due to their established safety in the food industry (generally regarded as safe—GRAS) and their role as probiotics with beneficial health effects when consumed in adequate amounts [19]. Within the LAB category, *Lactobacillus brevis* and *Lactobacillus plantarum* have emerged as particularly noteworthy and practical strains capable of producing elevated levels of GABA. Consequently, GABA has made a substantial contribution to the research and development of GABA-fortified foods.

Glycine is another amino acid that is known to improve sleep. Studies have shown that glycine activates N-methyl-D-aspartate (NMDA) receptors in the suprachiasmatic nucleus (SCN). In animals, it is known that activated NMDA receptors decrease nocturnal core body temperature, which increases the proclivity for sleep [20]. In humans, it has been reported that glycine taken before bedtime reduced daytime sleepiness, shortened polysomnographic sleep onset latency (SOL), and improved subjective sleep quality and memory task performance in individuals with sleep complaints [21]. Moreover, L-serine, a precursor for glycine, has been shown to improve sleep quality [22].

GABALAGEN (GBL) is a fermented collagen enriched with GABA. Through two consecutives fermentation process involving specialized Lactobacillus strains (*L. brevis*-BJ20 and *L. plantarum*-BJ21) for GABA production, collagen is further hydrolyzed into a lower molecular weight and GABA is produced as the by-product. Although no studies have directly investigated the effect of GBL on sleep, the high concentrations of GABA and glycine in GBL indicate a potential positive impact on sleep efficacy for individuals with sleep complaints. This hypothesis is grounded in the established roles of GABA and glycine in promoting relaxation and sleep, as documented in existing literature regarding the effects of these amino acids on the central nervous system [12,20]. Furthermore, GBL may be more effective in comparison to individual GABA or glycine due to its synergetic effect on factors associated with sleep regulation.

In this study, we evaluated the therapeutic potential of GBL for mitigating sleep disturbances by utilizing a pentobarbital-induced sleep-related animal models in rats. The primary aim of this study was to elucidate the effects of GBL through receptor binding assays and validate its efficacy in improving sleep by conducting experiments on animal sleep models.

## 2. Results

### 2.1. GABA Contents and Amino Acid Composition in GBL

**Figure 1:**
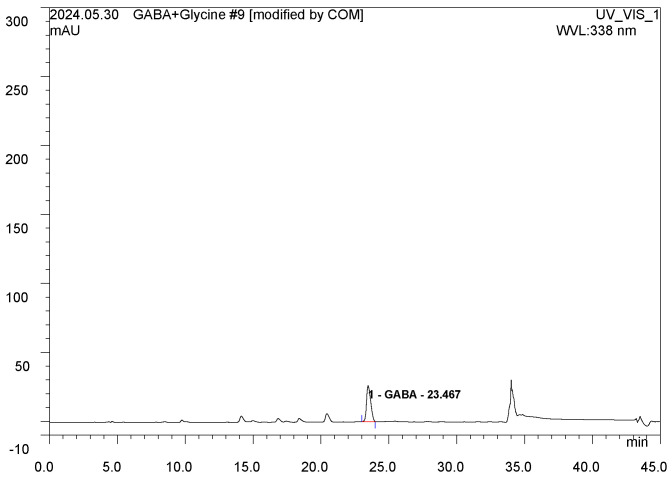
GBL chemical analysis. HPLC chromatograph of GABA in GBL.

To determine the GABA content in GBL, HPLC analysis was performed. The presence of GABA after fermentation was confirmed by comparing the retention times of the standard and GBL (Figure 1). The HPLC analysis results indicated that the average GABA content in GBL was 11.39%. Additionally, the amino acid composition of GABALAGEN showed significantly increased levels of threonine, aspartic acid, serine, glutamic acid, glycine, alanine, valine, leucine, arginine, proline, carnosine, and ornithine compared to the initial collagen, as presented in Table 1.

### 2.2. Binding Affinity GBL to 5-HT_2C_ Receptor

**Figure 2:**
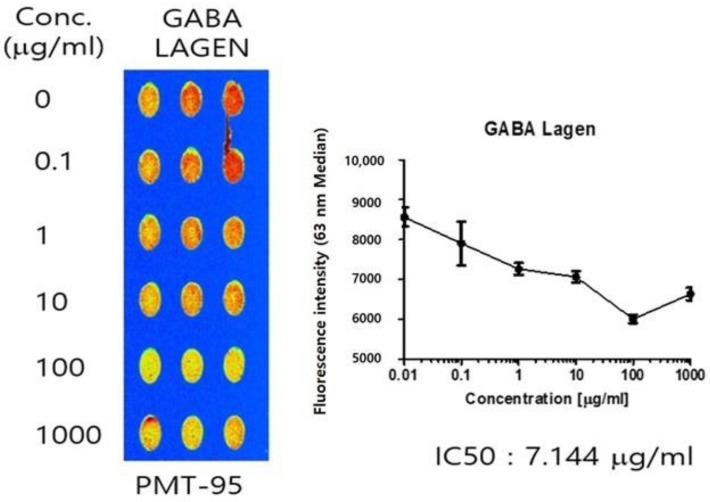
GBL showed a high binding affinity to 5-HT_2C_ receptor, supporting its sedative potential. Dose-response curve and IC50 of GBL in the 5-HT_2C_ receptor binding assay. GBL was added to 5-HT_2C_ receptors in a dose-dependent manner, revealing an IC50 value of 7.144 µg/mL. The concentration of 5-HT_2C_ receptors was 50 μg/mL, and the concentration of Cy5-labeled tryptamine was 500 μM.

The study demonstrated that GBL exhibits robust binding activity to the 5-HT_2C_ receptor, characterized by its efficacy as a 5-HT_2C_ receptor agonist. Notably, the inhibitory concentration (IC50) of GBL was determined to be 7.144.911 µg/mL (Figure 2). GBL exhibited a pronounced inhibitory effect on the interaction with the 5-HT_2C_ receptor.

### 2.3. Effect of GBL on EEG Sleep Architecture and Profile

**Figure 3:**
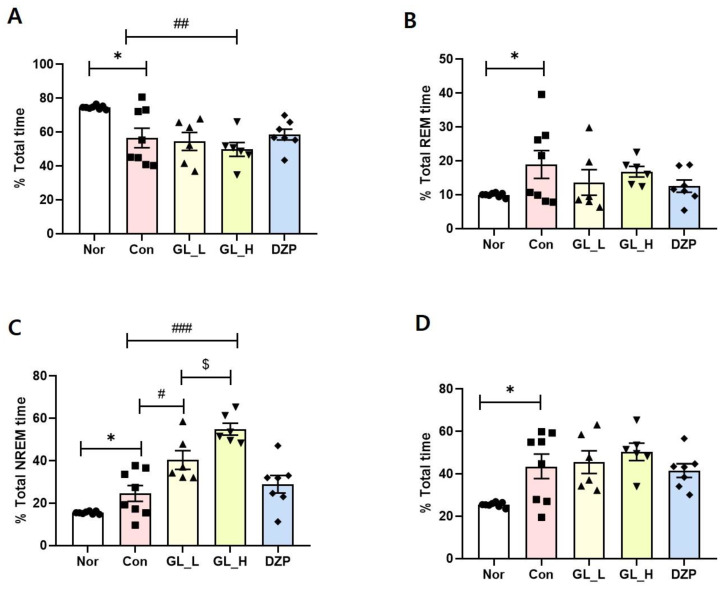
GBL enhanced NREM sleep and reduced wake time in pentobarbital-induced rat model. Changes in the percentage of wake (**A**), REM sleep (**B**), NREM sleep (**C**), and total sleep (**D**) during the dark phase are depicted in the GBL-treated groups. The data represent the mean ± SEM of the percent time spent in the sleep–wake state. * *p* < 0.05 vs. Nor, ^###^ *p* < 0.001, ^##^ *p* < 0.01, ^#^ *p* < 0.05 vs. Con, ^$^ *p* < 0.05 vs. GL_H; one-way ANOVA followed by Tukey’s LSD and *t*-test. The group labels are defined as follows. Nor: Normal group, untreated and naïve. Con: Control group, injected with pentobarbital and vehicle without any additional treatment. Nor (*n* = 8), Con (*n* = 8), GBL _L (*n* = 6), GBL _H (*n* = 6), DZP (*n* = 7).

The effect of GBL on EEG sleep architecture and profile was investigated. In the Con group, there was a significant reduction in wake time (*p* = 0.0217, η^2^ = 0.4091, Figure 3A) and a marked increase in REM (*p* = 0.0472, η^2^ = 0.2526, Figure 3B), NREM (*p* = 0.0307, η^2^ = 0.2920, Figure 3C), and total sleep (*p* = 0.0075, η^2^ = 0.4100, Figure 3D) compared to that of the Nor group. The GBL_L-treated group showed dramatically upregulated NREM (*p* = 0.0064, η^2^ = 0.4753, Figure 3C) compared to that of the Con group. The GBL_H-treated group showed significantly increased NREM (*p* < 0.001, η^2^ = 0.6349, Figure 3C) compared to that of the Con group. The DZP-treated group exhibited significantly increased wake time (*p* = 0.0463, η^2^ = 0.1221, Figure 3A) and upregulated NREM compared to that of the GBL_L and GBL_H groups (*p* < 0.001, η^2^ = 0.6349, Figure 3C). GBL_H produced notably enhanced sleep efficacy compared to GBL_L (*p* < 0.05).

### 2.4. Effect of GBL on 5-HT Level in the Serum

**Figure 4:**
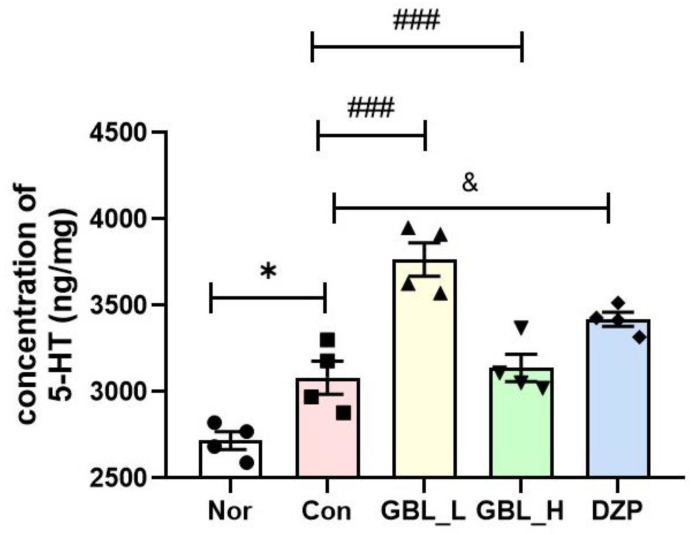
Effects of GBL on 5-HT level in the serum. * *p* < 0.05 vs. Nor; ^###^ *p* < 0.001 vs. Con; ^&^ *p* < 0.05 vs. DZP, one-way ANOVA followed by Tukey’s test. Nor (*n* = 4), Con (*n* = 4), GBL_L (*n* = 4), GBL _H (*n* = 4), DZP (*n* = 4).

The concentration of brain 5-HT was measured in the GBL-treated groups (Figure 4). The Con group had significantly increased 5-HT levels in the serum compared to those of the Nor group (*p* < 0.001). The GBL-treated groups exhibited significantly increased 5-HT levels in the serum compared to those of the Con group and DZP group (*p* < 0.001). The DZP-treated group had significantly increased 5-HT levels in the serum compared to those of the Con group (*p* = 0.0177, η^2^ = 0.6360). These findings suggest that GBL may enhance sleep patterns by modulating the serotonergic system.

### 2.5. Effect of GBL on the Intensity of Orexin-Positive Cells in the Lateral Hypothalamus (LH)

**Figure 5:**
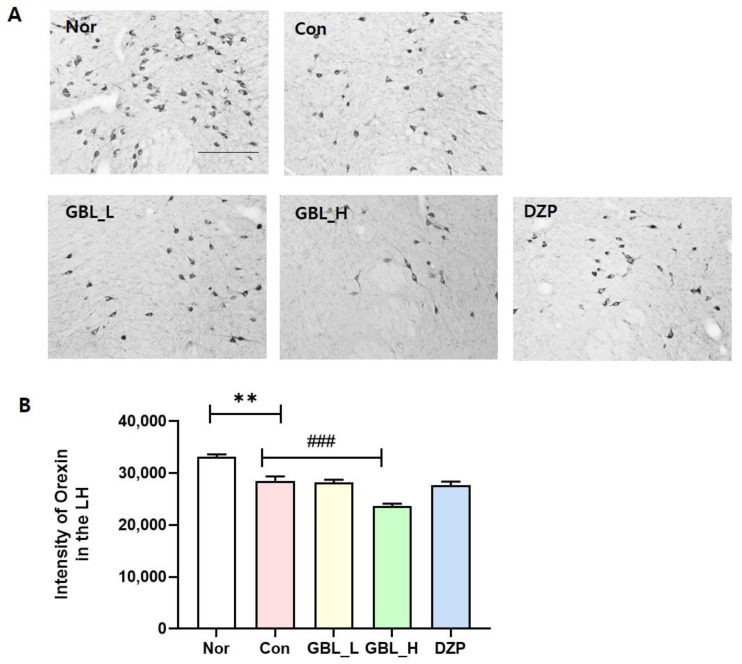
Impact of GBL on orexin-positive cells in the LH. (**A**) Photomicrographs illustrating orexin-positive cells in the LH. (**B**) Quantification of orexin-positive cells in the LH. ** *p* < 0.01 vs. Nor; ^###^ *p* < 0.001 vs. Con; one-way ANOVA followed by Tukey’s test. The scale bar represents 100 µm.

The orexin neurons within the LH are pivotal in governing the modulation of sleep-wake patterns. Pentobarbital administration instigates sleep by exerting inhibitory effects on the CNS, resulting in diminished orexin activity within the LH. We conducted orexin expression in the LH (Figure 5) and found that the numbers of orexin-positive cells were lower in the Con group than those in the Nor group (*p* = 0.0071, η^2^ = 0.1414). The GBL_H-treated group showed a dramatically reduced intensity of orexin-positive cells compared to the Con group (*p* < 0.001). These findings suggest that GBL has the potential to enhance sleep patterns by reducing arousal, as evidenced by a decrease in orexin expression.

### 2.6. Effect of GBL on 5-HT Level in the Dorsal Raphe Nucleus

**Figure 6:**
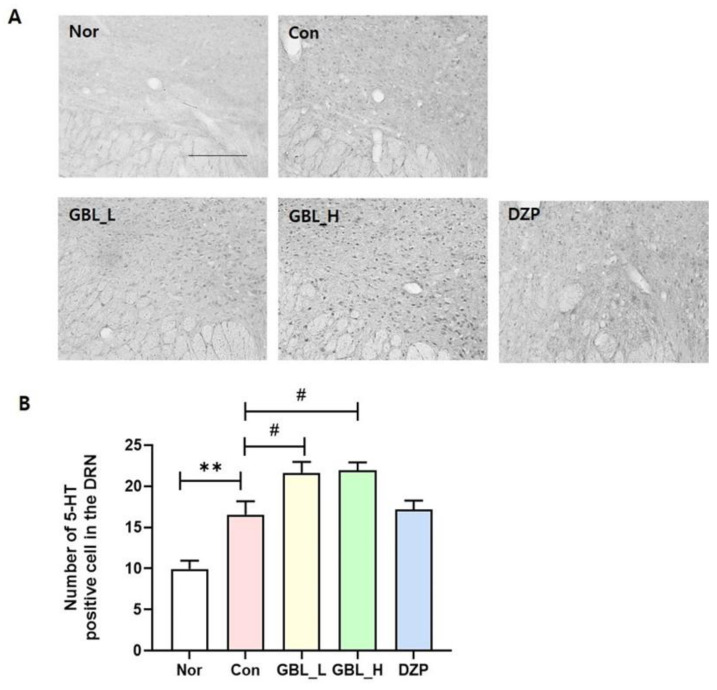
Impact of GBL on 5-HT-positive cells in the DRN. (**A**) Photomicrographs illustrating 5-HT-positive cells in the DRN. (**B**) Quantification of 5-HT-positive cells in the DRN. ** *p* < 0.01 vs. Nor; ^#^ *p* < 0.05 vs. Con; one-way ANOVA followed by Tukey’s test. The scale bar represents 100 µm.

The level of 5-HT in the brain was assessed in the GBL groups (Figure 6). The Con group showed dramatically upregulated levels of 5-HT in the DRN region compared to the Nor group (*p* = 0.0089). The GBL_L group and the GBL_H group showed significantly increased 5-HT immunoreactivity in the dorsal raphe nucleus (DRN) region compared to the Con group (*p* < 0.05, Figure 6).

## 3. Discussion

In the present study, GBL had sedative effects. In the screening test for GBL, it exhibited a binding activity to 5-HT_2C_ receptors. The administration of GBL_L resulted in a significant reduction in total wake time, accompanied by an increase in NREM time. The research shows that by administering GBL_H, NREM time is increased. GBL_H administration demonstrated a substantial decrease in orexin levels in the LH. Moreover, treatment with GBL_L showed a noteworthy increase in 5-HT levels in the serum.

Receptor binding analysis is a crucial tool for screening drug candidates, especially the 5-HT_2C_ receptor binding analysis that is widely used for screening calming and anxiolytic effects [23]. In the screening test for GBL, it exhibited the highest binding affinity to the 5-HT_2C_ receptor (IC50 value of 7.144 µg/mL). This result indicates that GBL exhibited a higher binding activity to 5-HT_2C_.

Pentobarbital has been employed as a sedative-hypnotic, anticonvulsant, and anesthetic, with varying effects depending on the dosage. Elevated doses of pentobarbital lead to anesthesia or profound central nervous system inhibition, while lower doses induce sedation [24]. To assess whether GBL has a synergistic effect on sleep with pentobarbital, we utilized a pentobarbital-induced sleep animal model, recording EEG. In NREM, the administration of pentobarbital increases the brainwave cycle, whereas in wakefulness, it decreases [25]. These EEG changes provide essential information for evaluating the impact of the drug on sleep and wakefulness. Similarly, in our study, the administration of pentobarbital in mice decreased wakefulness and increased NREM sleep time, inducing a sleep model [26]. These results suggest that pentobarbital plays a role in stabilizing sleep patterns by restoring wakefulness and NREM sleep times. In our study, GBL decreased wake time and increased total sleep time and NREM. Therefore, GBL exhibits the ability to enhance pentobarbital-induced sleep, supporting the notion that GBL contributes to the modulation of sleep stages.

The LH emerges as a crucial brain region intricately involved in the regulation of sleep-wake cycles [27]. Orexin neurons are characterized by wakefulness-promoting activity, reaching peak firing rates during wakefulness, and exhibiting the highest extracellular levels of orexin during wakefulness [28]. The LH coordinates sleep-promoting activities. By inhibiting REM sleep and, to a much less extent, promoting wakefulness, orexin helps maintain a balance between sleep and wakefulness. This emphasizes the importance of investigating the role of the LH and orexin in comprehending and addressing sleep-related disturbances. We found that GBL decreased expression of orexin-positive cells in the LH region. GBL_H may decrease orexin in the LH in a dose-dependent manner, leading to a reduction in arousal and potentially enhancing the quality of sleep.

Several studies have examined the role of GABA in sleep regulation, focusing on various brain regions [29]. The functions of the GABAergic system in these areas have highlighted their significant roles in the regulation of the sleep/wake cycle. While VLPO neurons may predominantly promote wakefulness, specific subsets of GABAergic neurons induce sleep [30]. Recent studies suggest that selective excitation of the POA^GABA^ to LH pathway increases wakefulness [31]. Previous research has also implicated VTA^GABA^ neuron terminals in the LH in regulating GABA’s role in sleep [29]. Recent advances in understanding the functions of the GABAergic system in brain regions have further clarified their significant roles in sleep/wake regulation. These findings can provide a crucial foundation for understanding the complex neurobiological mechanisms through which GBL affects sleep impairment and sleep disorder.

Numerous neurotransmitters, including GABA, 5-HT, norepinephrine, and dopamine, play vital roles in regulating various brain nuclei to control the transition between wakefulness and sleep [32]. Sleep is regulated by neurons that release classical fast neurotransmitters, including GABA. During the investigation, it was discovered that GBL aids in sleep by activating a pathway associated with 5-HT. The importance of 5-HT in regulating sleep is evident from studies that show that animals with reduced 5-HT production have longer wakefulness periods and less sleep time [33]. Many research studies also indicate that increasing 5-HT levels in the brain can extend sleep time in animals. These observations align consistently with the outcomes of our study. Consequently, we confirmed that GBL has the ability to enhance pentobarbital-induced sleep activity by observing an increment in 5-HT in the serum.

GABA-rich foods have recently been recognized as effective and safe bioactive substances. High doses of GABA-containing black tea have been shown to reduce anxiety [34]. In a previous study, a fermented rice-germ extract containing GABA normalized caffeine-induced sleep disorders [35]. Moreover, in another study, the combination of GABA and L-theanine not only reduced sleep latency but also prolonged sleep duration in a pentobarbital-induced sleep model. Additionally, in the caffeine-induced arousal model, combined GABA and l-theanine is an attractive NREM sleep-promoting regimen as it increases delta wave oscillations [18]. Therefore, it has been suggested that GABA-enriched foods may have beneficial effects on sleep behaviors.

In our study, we tested this hypothesis and found that administration of GBL produced a dose-dependent decrease in wake time and an increase in NREM sleep with pentobarbital. GBL_H demonstrated significant hypnotic activity in both wakefulness and NREM sleep compared to the CON group. Pentobarbital-induced sleep behaviors were notably enhanced by DZP (10 mg/kg), a medication commonly used to treat psychiatric conditions such as anxiety and insomnia. DZP enhances GABA activity by binding to the benzodiazepine site on the GABAA receptor, resulting in behavioral effects.

Our findings suggest that GBL is more effective than DZP in enhancing sleep behaviors. These differential effects may be attributed to the fact that while DZP enhances GABA effects solely through binding to the benzodiazepine site on the GABAA receptor, GBL has been demonstrated to bind to 5-HT_2C_ receptors, potentially influencing the serotonergic system. However, there is currently no direct evidence of GBL binding to GABAA-BZD receptors, and further investigation is required to confirm this interaction. Although the interaction of GBL with 5-HT_2C_ receptors is supported by the data, its potential binding to GABAA-BZD receptors has not been confirmed and necessitates further research to elucidate this mechanism.

In 5-HT_2_ receptor binding assays, GBL binds very highly and competitively to 5-HT binding sites on receptors. An agonist or antagonist activity against 5-HT_2_ serotonin receptors were not tested in the present study. However, we assumed that GBL may be a 5-HT_2C_ receptor antagonist since it was very highly competitive with tryptamine, a basic neurotransmitter, confirming its selectivity for serotonin receptors. Therefore, 5-HT_2C_ may have antagonistic effects on behavior, and we assumed its efficacy as a 5-HT_2C_ receptor agonistic affecting behavior. Previous studies have shown that antagonists of the 5-HT_2C_ receptor increase NREM sleep [36], indicating effectiveness in promoting sleep. Consistent with this, our results demonstrate that GBL enhances NREM sleep, thus improving sleep quality.

As far as we have known, there is no direct interaction or influence of GABA on 5-HT_2C_ receptors. They operate independently, each responding to their respective neurotransmitter. However, it should be noted that neurotransmitter systems in the brain can interact indirectly through complex neural circuits, but these interactions involve broader networks rather than direct receptor-to-receptor interactions between GABA and 5-HT_2C_ receptors. For example, there is evidence that GABA can influence serotonin receptors, including 5-HT receptors such as 5-HT1A and 5-HT_2A_ [37]. Therefore, it is possible that GABAlagen increases in GABA-activated serotonin systems through 5-HT_2C_ receptors, and these connections can modulate the activity of serotonergic neurons and thereby influence the release of serotonin.

In addition, GABA may can indirectly affect the activity of 5-HT_2C_ receptors. It is known that increased GABAergic inhibition via GABAA receptors can enhance the activity of 5-HT_2C_ receptors in the hypothalamus, influencing functions such as sleep regulation, appetite suppression, and temperature regulation. Therefore, the interaction between the GABAergic system and serotonin receptors like 5-HT_2C_ plays a crucial role in sleep control.

The involvement of GABA in GBL’s influence on sleep is supported by several key points: (1) the high GABA content in GBL, (2) the established effects of GABA on sleep regulation, and (3) the effects of known GABAA receptor modulators such as pentobarbital and diazepam. GABA is widely recognized for its role in promoting sleep and reducing wakefulness, and its presence in GBL may contribute to the compound’s sedative properties. This is consistent with the action of other sleep-promoting agents, such as pentobarbital and diazepam, which enhance GABAA receptor activation. Given these similarities, it is plausible that GBL exerts its effects through a related pathway, modulating GABAergic activity to influence sleep architecture.

This study had some limitations. First, the number of subjects included was too small. However, we believe that the quality of the EEG signal was good enough since we recorded EEG through electrode implantation, and it was not contaminated by motion artifacts. Second, the specific mechanisms underlying GBL’s effects on wake/REM/NREM sleep were not explored, necessitating further research. Third, the long-term effects and safety of GBL when used alone have not been thoroughly evaluated. Fourth, the study was conducted via animal models, and therefore, the results may not be directly applicable to humans. Lastly, we need to conduct functional assays to test whether the activity is agonist or antagonist in order to comprehensively understand GBL’s pharmacological profile using a variety of 5-HT_2C_ agonists and antagonists in further study. To address these limitations, future research should evaluate various dosages and long-term effects, and conduct clinical trials to investigate the effects of GBL on human subjects.

## 4. Conclusions

Based on the observed sleep improvement effects of GBL in rodents, our objective is to propose it as a potential sleep aid or treatment for sleep disorders in humans. In this study, we employed receptor binding assays and analyzed key parameters, including EEG, orexin, and 5-HT, to investigate the impact of GBL on sleep in a rodent model. However, these analyses only verified partial effects of GBL. In the pentobarbital-induced sleep model, GBL reduced wakefulness and increased total sleep. Notably, the efficacy of GBL was found to be superior to that of the conventional sleep aid DZP. Additionally, orexin expression levels decreased, and 5-HT expression levels decreased. The specific mechanisms underlying GBL’s effects on wake/REM/NREM sleep were not explored, necessitating further research.

## 5. Material and Methods

### 5.1. Preparation of GBL

GABALAGEN(GBL) was acquired from Marine Bioprocess Co., Ltd. (Busan, Republic of Korea). Before fermentation, fish collagen from Geltech Co., Ltd. (Busan, Republic of Korea) was hydrolyzed at 55 °C ± 2 °C for 12 h with prozyme (Bisionbiochem Co., Ltd., Seoul, Republic of Korea). GBL production required two consecutive fermentations via *Lactobacillus brevis* BJ20 (accession No. KCTC 11377BP) and *Lactobacillus plantarum* BJ21 (accession No. KCTC 18911P). Seed media composed of 3% yeast extract (Choheung, Ansan, Republic of Korea), 1% glucose (Choheung, Ansan, Republic of Korea), L-glutamic acid 1% (Samin Chemical, Siheung, Republic of Korea), and 95% water was sterilized for 15 min at 121 °C before being inoculated with 0.002% BJ20 and 0.002% *Lactobacillus plantarum* BJ21 and cultured for 24 h at 37 °C separately. For first fermentation, 10% (*v*/*v*) of the *Lactobacillus brevis* BJ20 cultured seed media was fermented in a fermentation medium (yeast extract 2%, glucose 0.28%, hydrolyzed fish collagen 29%, L-glutamic acid 5.5%, water 63.22%) at 37 °C for 24 h. Then, 10% (*v*/*v*) of BJ21 cultured seed medium was added and fermented at 37 °C for another 24 h. The fermentation medium was sterilized and spray-dried to prepare GBL powder samples.

### 5.2. High-Performance Liquid Chromatography (HPLC) Analysis

#### 5.2.1. Chemical and Reagent

GABA and sodium acetate (50 mM, pH 6.5) were obtained from Sigma-Aldrich (St. Louis, MO, USA). HPLC-grade acetonitrile, methanol, and distilled water (DW) were procured from Samchun Pure Chemical Co., Ltd. (Pyeongtaek, Republic of Korea). The hydrochloric acid solution was provided by Biosesang (Seongnam, Republic of Korea). Borate buffer (0.4 N in water, pH 10.2; Agilent P/N 5061-3339) and o-phthaldialdehyde reagent (10 mg/mL, Agilent P/N 5061-3335) were sourced from Agilent Technologies (Palo Alto, CA, USA). Acetic acid was acquired from Junsei Chemical Co., Ltd. (Tokyo, Japan).

#### 5.2.2. Standard Solution and Sample Preparation

A 0.1 g standard sample was dissolved in 100 mL of DW in a volumetric flask to prepare a standard solution. The solution was then filtered through a polytetrafluoroethylene syringe filter (25 mm/0.2 μm) and kept at −80 °C. For the 5% aqueous sample solution, 5 g of GBL was dissolved in DW in a 100 mL volumetric flask and similarly filtered through a polytetrafluoroethylene syringe filter.

#### 5.2.3. HPLC Analysis Method

A Dionex U3000 series HPLC system (Thermo Fisher, Waltham, MA, USA) equipped with an ultraviolet (UV) detector was used, and the flow rate was reduced to 1 mL/min. The samples were analyzed using UV–vis spectrophotometry at a wavelength of 338 nm. The amount of GABA in GBL was calculated using the following equation:

$$\mathrm{Substance}\;(\mathrm{mg}/g)=\frac{\mathrm{measurement}\;(\mathrm{mg}/\mathrm{mL})\;\times \;\mathrm{dilution}\;\mathrm{factor}}{\mathrm{amount}\;(g)}\times 100(\mathrm{mL})$$

#### 5.2.4. 5-HT_2C_ Receptor Binding Assay

The 5-HT_2C_ receptor (serotonin 5HT_2C_ membrane preparation in HEK293 cells) was procured from PerkinElmer (Waltham, MA, USA). The protein chip was obtained from Proteogen, and Cy5-labeled tryptamine was acquired from Peptron. The stock buffer for the 5-HT_2C_ receptor comprised 50 mM Tris-HCL (pH 7.4), 0.5 mM EDTA, 10 mM MgCl_2_, and 10% sucrose. The 5-HT_2C_ receptor binding assay buffer comprised 50 mM Tris-HCl, 10 mM MgCl, 1 mM EDTA, and 0.1% bovine serum albumin (BSA) at pH 7.4. The 5-HT_2C_ receptor (50 µg/mL) was immobilized on the protein chip, serving as a substrate to capture protein, for 16 h at 4 °C. After double washing in 0.05% phosphate-buffered saline containing 0.2% Triton X-100 (PBST) for 10 min and drying with nitrogen gas (N_2_), the protein chip underwent a 1 h blocking step at room temperature using 3% BSA. Following three washes with PBST and drying, Cy5-labeled tryptamine (500 µM, with 30% glycerol in PBS as a buffer) and GBL (using 30% glycerol in PBS as a buffer) were applied to the protein chip and incubated for 1 h at 37 °C. Subsequently, the protein chip was rinsed with PBST and DW and dried under a stream of N_2_ gas. GBL, dissolved in ethanol and diluted to the desired concentration using PBS, covered a concentration range from 1000 µM to 15.625 µg/mL. Tryptamine was used as a negative control. GBL was used to evaluate the sedative effect of the 5-HT_2c_ receptor binding assays.

### 5.3. Animals

Adult male Sprague-Dawley rats were obtained from Samtako (Osansi, Gyeonggi-do, Republic of Korea). These animals were accommodated in a climate-controlled environment with temperatures maintained between 20 °C and 25 °C and humidity levels set at 45% to 65%, following a 12 h light and 12 h dark cycle (lights on at 8 a.m.). Food and water were provided ad libitum throughout the study. The laboratory animals were handled and treated in compliance with the guidelines outlined by the Ministry of Food and Drug Safety (MFDS) National Institute of Toxicological Research, as per the standards for laboratory animal care and usage. Rats were randomly assigned to five groups: untreated, naïve (Nor, *n* = 8), pentobarbital injected with vehicle (CON, *n* = 8), pentobarbital injected along with 100 mg/kg GBL (GBL_L, *n* = 6), pentobarbital injected along with 250 mg/kg GBL (GBL_H, *n* = 6), and pentobarbital injected along with 10 mg/kg DZP (DZP, *n* = 7). Diazepam is widely recognized as a sedative drug and was used as a positive control in this experiment.

### 5.4. EEG Surgery

The subjects were divided into five groups: normal (Nor; *n* = 7), control (con; *n* = 8), low-dose GBL-treated (GBL_L; *n* = 6), high-dose GBL-treated (GBL_H; *n* = 7), and diazepam-treated (DZP; *n* = 7). Electroencephalogram (EEG) electrodes were surgically implanted to facilitate polygraphic recordings, following the guidelines delineated in the Paxinos and Watson stereotaxic atlas [38]. Surgical anesthesia was induced with intraperitoneal pentobarbital (40 mg/kg). Subsequently, the rats were subjected to chronic implantation of a head mount. The transmitter body was subcutaneously placed off the midline, posterior to the scapula, secured to the skin, and stabilized using three sutures. Skull-mounted electrodes were secured with screws and dental cement. All surgical interventions were performed using a stereotaxic methodology in an aseptic environment. Postoperatively, each rat was allowed a 7-day recovery period in separate transparent enclosure.

### 5.5. Methodology of EEG Recording

Following the recovery phase, the rats were acclimated to the recording settings before testing. GBL_L, GBL_H, and DZP were prepared in 0.9% saline (GBL_L concentration = 100 mg/kg, GBL_H concentration = 250 mg/kg, DZP concentration = 10 mg/kg) and orally administered for five consecutive days before the EEG recording commenced. The oral administration of saline, GBL_L, GBL_H, and DZP was performed 45 min prior to administration of pentobarbital. Then, after administration of saline, GBL_L, GBL_H, and DZP, sleep was induced using pentobarbital at a sleep-inducing concentration (42 mg/kg, i.p.) and the EEG recording sessions were performed. The recordings were initiated at 8:00 p.m., capturing 12 h of EEG recordings and activity in all subjects. The cortical EEG signals were amplified (×100), filtered through a low-pass filter at 100 Hz, digitized at a sampling rate of 200 Hz, and recorded using a PAL-8200 data acquisition system from Pinnacle Technology Inc. The recordings were performed at a chart speed of 25 mm/s.

#### EEG Data Analysis

The SleepSign Ver. 3 software (Kissei Comtec, Nagano, Japan) automatically classified sleep-wake states into three categories: wakefulness (wake), rapid eye movement (REM) sleep, and NREM sleep. Sleep latency was defined as the duration from the administration of the sample to the onset of the initial uninterrupted NREM sleep episode, lasting a minimum of 2 min and not interrupted by more than six 4 s epochs that were not scored as NREM sleep.

### 5.6. Measurement of 5-HT Concentration in the Serum

After the behavioral tests, cardiac blood was collected and centrifuged to separate the serum. The obtained samples were stored at −80 °C until the enzyme-linked immunosorbent assay (ELISA kit; Enzo Life Sciences, Farmingdale, NY, USA) was performed to measure serum 5-HT.

### 5.7. Immunohistochemistry

After transcranial perfusion, the brains were dissected, then post-fixed in 4% formaldehyde overnight, and placed in 30% sucrose solution for 24 h at 4 °C. Samples were cut into 30 μm thickness and the sections were kept at −20 °C. The brain sections were washed in PBS three times for 10 min and then quenched for 10 min at RT 3% H_2_O_2_ in PBS. Samples were rinsed in PBS three times for 10 min and blocked for 1 h at RT 0.2% triton X-100 (Sigma) and 1.5% bovine serum albumin (BSA) (Sigma, Burlington, MA, USA) in PBS. Sections were rinsed in PBS containing 0.5% BSA three times for 10 min.

Primary rabbit polyclonal antibodies against orexin and 5-HT (Abcam, Cambridge, MA, USA) were diluted to 1:800. The sections underwent a 12 h incubation at 4 °C with constant agitation. After rinsing with PBST, a 2 h incubation at room temperature was performed using a biotinylated goat anti-rabbit antibody (Vector Laboratories, Inc., Burlingame, CA, USA) diluted to 1:200 in PBST with 2% *v*/*v* normal goat serum. Subsequently, the sections were exposed to an avidin-biotin-peroxidase complex reagent (Vector Laboratories) for 2 h at room temperature. After further rinsing with PBST, the tissues were developed using a DAB substrate kit (Vector Laboratories). The final steps included mounting the sections on slides, air-drying, and covering them for microscopic observation. The images were captured using a DP2-BSW imaging system (Olympus, San Jose, CA, USA). Cells testing positive for orexin were counted on a grid that was placed on LH. The number of cells was counted at a magnification of 100×, using a rectangular microscopic grid measuring 200 × 200 μm^2^.

### 5.8. Statistical Analysis

All statistical analyses were conducted using SPSS (IBM^®^ SPSS^®^ Statistics Ver. 23 Chicago, IL, USA). For multiple comparisons, behavioral data were analyzed using one-way analysis of variance (ANOVA) and *t*-test. Bonferroni’s post hoc test was used to identify significant differences among groups. The level of significance was set at *p* < 0.05.

## Figures and Tables

**Table 1:** Amino acid composition in GBL.

Free Amino Acid	Collagen	GBL
aspartic acid	25.8	83.9
threonine	10.2	78.1
serine	11.7	118.5
glutamic acid	0	64.3
glycine	87.4	386.7
alanine	145	711.7
valine	176.6	271.6
leucine	31.4	238.2
arginine	130.8	160.1
proline	0.4	32.7
a-amino-n-butyric acid	75.7	379.6
carnosine	34.1	177.4
ornithine	24.1	77.4
γ-amino-n-butyric acid	56.9	9471.9

## Data Availability

The data used to support the findings of this study are available from the corresponding author upon request.
